# Low Request of Antibiotics from Patients with Respiratory Tract Infections in Six Countries: Results from the Happy Audit Study

**DOI:** 10.3390/antibiotics2040477

**Published:** 2013-11-19

**Authors:** Carl Llor, Lars Bjerrum, Eva Lena Strandberg, Ruta Radzeviciene, Anatoliy Reutskiy, Lidia Caballero

**Affiliations:** 1 Primary Healthcare Centre Jaume I, University Rovira i Virgili, Tarragona 43205, Spain; 2 Section of General Practice and Research Unit for General Practice, Department of Public Health, University of Copenhagen, Copenhagen 1014, Denmark; E-Mail: lbjerrum@sund.ku.dk; 3 Department of Clinical Sciences, General Practice/Family Medicine, Lund University, Malmö 205 02, Sweden; E-Mail: Eva-Lena.Strandberg@med.lu.se; 4 Family Doctors Centre Mano šeimos gydytojas, Klaipeda 91220, Lithuania; E-Mail: ruta.r@takas.lt; 5 Association of Family Doctors, Kaliningrad 236044, Russia; E-Mail: areutskiy@gmail.com; 6 Misiones Association of General Family Medicine, Posadas 3300, Argentina; E-Mail: lijo@arnet.com.ar

**Keywords:** audit, respiratory tract infections, antibiotics, demand

## Abstract

A total of 59,535 patients with respiratory tract infections were registered in the Happy Audit project, an audit-based, before-and-after study conducted in primary care centres of six countries (Argentina, Denmark, Lithuania, Russia, Spain, and Sweden) in 2008 and 2009. An antibiotic was explicitly requested by the patient in 1,255 cases (2.1%), with a great variation across countries ranging from 0.4%–4.9%. Antibiotics were significantly more often prescribed to patients requesting them compared to those who did not (64% *vs*. 28%; *p* < 0.001). Patients with acute exacerbations of chronic bronchitis/chronic obstructive pulmonary disease were most likely to request antibiotics while those with common colds were least likely (3.9% *vs.* 1.2%, respectively). The presence of tonsillar exudates and dyspnoea were more commonly associated with a demand for antibiotics. Even though physicians very often perceive that patients demand an antibiotic, the results of this study clearly show that patients only request antibiotics in a low percentage of cases. Patients were most likely to request antibiotics when they had symptoms of lower respiratory tract infections and when they came with more severe symptoms. Furthermore, there were considerable differences between countries, suggesting that the different backgrounds and traditions largely explain this variability in patients’ requests for antibiotics.

## 1. Introduction

Antibiotics are among the most frequently used pharmaceuticals today. Since the development of penicillin, antibiotic use in all parts of the health care system has significantly contributed to reducing the likelihood of dying from an infectious disease [1]. However, consumption tends to deplete the efficacy of the antibiotic in combating bacterial infections because the bacteria develop resistance to the antibiotic [2]. As a result, one of the primary strategies to prevent and control the emergence and spread of resistant organisms is to reduce the selective pressure of overuse and misuse of antibiotics in human medicine.

The perceptions of general practitioners (GP) regarding patient expectations for a prescription are one of the strongest predictors of prescribing, and this is even more so for antibiotic therapy [3]. When patients expect antibiotics they are more likely to be prescribed [4], and the same occurs when physicians perceive that patients expect antibiotics [5]. However, physicians are not very good at determining whether the patients actually expect antibiotic therapy [6]. Furthermore, there is compelling evidence that not providing antibiotics does not affect the patients’ satisfaction with the visit. Patient dissatisfaction has been shown to be more significantly related only to poor communication during the visit [6].

Despite the numerous studies on patients’ expectations and physicians’ perceptions and their impact on the prescription of antibiotics for respiratory tract infections, only few studies have addressed the explicit request for antibiotics by patients during the physician-patient consultation. GPs often prescribe an antibiotic to fulfil patient demands in an attempt to satisfy the patient, and a satisfied patient is a return customer, more likely to feel that the physician has appropriately addressed his or her needs. GPs very often feel uncomfortable when coping with this demand, making the prescription of an antibiotic more likely compared to situations in which this demand does not occur. Britten suggested that doctors may use a prescription to close difficult consultations [7]. It takes longer to explain to the patient why you are not prescribing antibiotics than it does to prescribe an antibiotic.

A prospective, non-randomised before-after study was performed in primary care clinics in five European countries (Denmark, Lithuania, Russia, Spain, and Sweden) and Argentina, as a part of the Happy Audit project, a study financed by the European Commission. The main objective of the Happy Audit was to strengthen the surveillance of respiratory tract infections in primary health care through the development of intervention programmes targeting GPs [8]. The aim of this study was to evaluate the number and the characteristics of patients who explicitly requested an antibiotic during the consultation. 

## 2. Results

### 2.1. Descriptives

**Figure 1:**
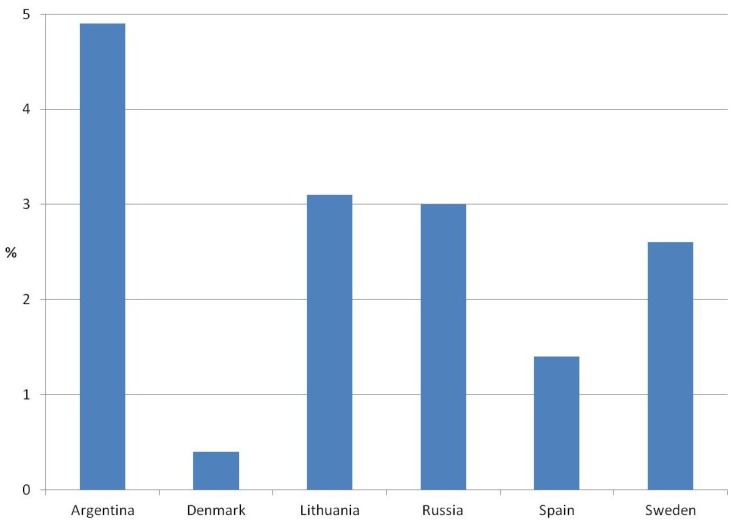
Patients with a demand for antibiotics in the Happy Audit study in relation to country.

A total of 59,535 contacts with respiratory tract infections were observed during the two years and of these, 1,255 cases requested the prescription of an antibiotic (2.1%). The mean age was 35 years with a range from 0–101 years, and 29% were younger than 18 years. As shown in Figure 1, an important variation was found across the countries, being higher in Argentina with 4.9% of the patients explicitly requesting an antibiotic and lowest in Denmark, where only 0.4% of the patients demanded the prescription of an antibiotic. Antibiotics were significantly more often prescribed to patients requesting them (799 patients, 64%) compared to those who did not (16,305 contacts, 28%; *p* < 0.001).

### 2.2. Characteristics of Patients Requesting Antibiotics

Patients requesting antibiotics were slightly younger than those who did not (32 *vs.* 35 years; *p* < 0.001). Patients younger than 18 years more frequently requested antibiotics than those who were older (2.6% *vs.* 1.9%, *p* < 0.001). No statistically significant differences were observed in the request for an antibiotic by gender, as shown in Table 1. The number of days with symptoms before contacting the GP was also similar between the two groups (4.3 *vs.* 4.1 days) with no statistical differences being found. As far as symptoms and signs were concerned, patient demand for antibiotics was highest with the presence of tonsillar exudates, being observed in 4.9% of the cases, followed by the presence of breathlessness (4.0%). On the other hand, the presence of cough was only associated with 2.1% of antibiotic requests.

**Table 1:** Characteristics of the group of patients (n = 59,535) with respiratory tract infections included in the Happy Audit study and the group of patients that explicitly requested antibiotics.

	All patients with respiratory tract infections, n (%)	Patients that explicitly requested antibiotics, n (%; 95% CI)
Gender		
- Male	26,157 (43.9)	561 (2.1; 2.2–2.3)
- Female	33,378 (56.1)	694 (2.1; 1.9–2.2)
Symptoms and signs		
- Fever	25,735 (43.2)	710 (2.8; 2.6–3.0)
- Cough	44,447 (74.7)	934 (2.1; 2–2.2)
- Purulent ear discharge	1,280 (2.1)	34 (2.7; 1.8–3.5)
- Painful swallowing	20,981 (35.2)	565 (2.7; 2.5–2.9)
- Tonsillar exudates	4,143 (7.0)	203 (4.9; 4.2–5.6)
- Tender cervical glands	4,857 (8.2)	191 (3.9; 3.4–4.5)
- Dyspnoea	5,592 (9.4)	222 (4.0; 3.5–4.5)
- Increased sputum production	9,947 (16.7)	320 (3.2; 2.9–3.6)
- Purulent sputum	4,466 (7.5)	133 (3.0; 2.5–3.5)
Aetiology suspected by the physician		
- Viral aetiology	41,338 (69.5)	805 (1.9; 1.8–2.1)
- Bacterial aetiology	14,552 (24.4)	373 (2.6; 2.3–2.8)
Diagnosis		
- Common cold	22,151 (37.2)	265 (1.2; 1.1–1.3)
- Acute otitis media	2,112 (3.5)	51 (2.4; 1.8–3.1)
- Acute sinusitis	1,945 (3.3)	32 (1.6; 1.1–2.2)
- Acute pharyngitis	7,241 (12.2)	189 (2.6; 2.2–3.0)
- Acute tonsillitis	4,643 (7.8)	149 (3.2; 2.7–3.7)
- Acute bronchitis	6,935 (11.6)	243 (3.5; 3.1–3.9)
- Pneumonia	2,063 (3.5)	26 (1.3; 0.7–1.7)
- Exacerbations of CB/COPD	2,073 (3.5)	80 (3.9; 3.0–4.7)
- Influenza	5,469 (9.2)	105 (1.9; 1.6–2.3)
- Other respiratory tract infections	3,959 (6.6)	95 (2.4; 1.9–2.9)

CI = confidence interval; CB = chronic bronchitis; COPD = chronic obstructive pulmonary disease.

Patients with acute exacerbations of chronic bronchitis/chronic obstructive pulmonary disease frequently requested antibiotics (3.9%) followed by those with acute bronchitis (3.5%) (Table 1).

## 3. Discussion

This study has shown that antibiotics are only requested by a low percentage of patients when they attend the GP’s office with a respiratory tract infection. However, GPs prescribed antibiotics twice as frequently when patients expressed a demand compared to when they did not. Patients with more severe symptoms and signs and those with lower respiratory tract infections—acute exacerbations of chronic bronchitis/chronic obstructive pulmonary disease and those with acute bronchitis—were most likely to express a demand for antibiotics. This study has also demonstrated that antibiotic request significantly varies across countries.

This study has some limitations. The percentage of patients that requested antibiotics was very low in our study, being only 1.5%, which is much lower than in other studies such as that by Coenen *et al*. who reported 15% of patients requesting an antibiotic [3]. In a questionnaire-based study carried out in the United States, Kuzujanakis *et al*. found that 24% of parents gave responses suggesting a proclivity to demand antibiotics for their children [9]. In our study, approximately one third of the infections were due to the common cold and in these cases the request for an antibiotic was very low. GPs were requested to tick off the box of request only when patients expressed a demand for antibiotics in the consultation for the respiratory tract infection. This fact may actually explain why the percentage of patients demanding antibiotics was so low.

There have been many studies reporting that physicians often feel pressured by patients to prescribe antibiotics. A survey among 610 paediatricians found that 48% were pressured by parents to prescribe antibiotics for conditions not requiring antimicrobial therapy. Approximately one-third of the paediatricians admitted that they generally complied with such requests, even in cases they believed did not warrant antibiotics [10]. Studies of adult patients in family practice settings have also clearly demonstrated that physicians are much more likely to prescribe medication when they think the patient expects a prescription. The lack of correlation between a GP’s anticipation of a patient’s request and patient satisfaction has been similarly documented in a study of adult patients in primary care settings [11]. Again, physicians were found to prescribe antibiotics much more frequently for respiratory infections when they thought the patient expected antibiotic therapy [12]. The physicians in this study did have some ability to judge which patients actually expected to receive antibiotics. Nevertheless, receipt of an antibiotic prescription was found to be unrelated to patient satisfaction. In another paper, Welschen *et al*. observed in a questionnaire-based study that receiving information and reassurance was more strongly associated with satisfaction than an antibiotic prescription [13]. In another questionnaire-based study, 69% of Irish GPs felt themselves to be under pressure by patients’ demands for antibiotics [14]. 

A recently published paper studied the association between the expectation, hope and request for antibiotics with data from the GRACE (Genomics to combat resistance against antibiotics in community-acquired lower respiratory tract infections in Europe) study [15]. The authors found that antibiotics were expected by 45% of the patients, but they were only requested in 10% of the cases. Data were obtained with 5-point scales and only lower respiratory tract infections were recruited. In our study GPs were clearly instructed to state that patients demanded an antibiotic when they explicitly asked the physician to prescribe one, considering this variable as dichotomous (yes/no). One of the most striking results was the difference in antibiotic prescribing by the GPs depending on whether or not the patient expressed a demand. In fact, the GP probably felt more pressured to prescribe antibiotics when patients echoed this wish during the consultation. The patients who more commonly requested antibiotics were those with suspected bacterial respiratory tract infections and those with more severe symptoms. The diagnoses that were most linked to antibiotic request were those with lower respiratory tract infections. Curiously, only 1.3% of patients with a diagnosis of pneumonia explicitly asked their physicians for an antibiotic, maybe because they were already treated with this drug. Another observation of this study was the scarce association of demographic data, such as age and gender in the demand of antibiotics. Our study did not allow comparison of the influence of other variables such as comorbidity on antibiotic request, constituting another weakness of this study, since comorbid conditions were not included in the chart. 

Another finding that must be highlighted has been the different percentages of antibiotic demand depending on the country, being higher in the southern European countries and Argentina and much lower in the Nordic countries. This could mainly be explained by the different GPs’ habits, traditions and types of primary health care systems in the countries studied. The differences in the health care systems are clearly shown with the results of the two Nordic countries. In Sweden, patients with respiratory tract infections are usually seen by triage nurses, which could explain why Swedish patients demanded more antibiotics than the Danish counterparts when the antibiotic prescribing is lower in Sweden [16]. On the basis of these results, a strategy addressing a change in patients’ behaviour should be considered in the high antibiotic-requesting countries in order to reduce the overprescribing of antibiotics. In conclusion, this paper examined the characteristics of the patients who requested antibiotics for respiratory tract infections providing clues for achieving a more rational use of antibiotics for these infectious conditions. 

## 4. Experimental

### 4.1. Methods

Data were obtained from GPs in two Nordic countries (Denmark and Sweden), two Baltic Countries (Lithuania and Russia) and two Hispano-American countries (Spain and Argentina). The data were registered according to the methodology of the Audit Project Odense which follows a prospective self-registry methodology in which a simple reporting sheet is used [8]. All the participants were instructed to fill out a template including all the patients consulted with respiratory tract infections during a 3-week period, covering a total of 15 working days in the winter months of 2008 and 2009. On this sheet the physicians registered different concrete parameters of medical care, including the age and gender of the patient, the number of days of symptoms, presenting signs (fever, cough, purulent otorrhoea, odynophagia, tender cervical glands, tonsillar exudate, dyspnoea, increased sputum production, purulent sputum), diagnosis, aetiological suspicion (viral or bacterial), antibiotic treatment or not, and whether the patient requested an antibiotic or not.

### 4.2. Analysis

Descriptive analysis was carried out. In addition, chi-squared and Student’s t tests were used to compare proportions and means, respectively. Statistical significance was considered with a *p* value <0.05.

## 5. Conclusions

Patient expectation for a prescription has a recognized influence on GP prescribing habits, particularly in relation to the prescribing of antibiotics. In this audit-based study, however, the percentage of patients who explicitly requested an antibiotic was only 2.1%. Nonetheless, a great variation was observed across countries, depending more often on the severity of symptoms and the diagnosis of the patients than on demographic variables, with the demand for antibiotics being more likely among patients presenting symptoms of lower respiratory tract infections. 
